# Analysis of Yellow Striped Mutants of *Zea mays* Reveals Novel Loci Contributing to Iron Deficiency Chlorosis

**DOI:** 10.3389/fpls.2018.00157

**Published:** 2018-02-20

**Authors:** David Chan-Rodriguez, Elsbeth L. Walker

**Affiliations:** ^1^Plant Biology Graduate Program, University of Massachusetts Amherst, Amherst, MA, United States; ^2^Department of Biology, University of Massachusetts Amherst, Amherst, MA, United States

**Keywords:** iron, phytosiderophores, yellow stripe, maize, mutants

## Abstract

The micronutrient iron (Fe) is essential for photosynthesis, respiration, and many other processes, but it is only sparingly soluble in aqueous solution, making adequate acquisition by plants a serious challenge. Fe is a limiting factor for plant growth on approximately 30% of the world’s arable lands. Moreover, Fe deficiency in humans is a global health issue, affecting 1.62 billion people, or about 25% of the world’s population. It is imperative that we gain a better understanding of the mechanisms that plants use to regulate iron homeostasis, since these will be important targets for future biofortification and crop improvement strategies. Grasses and non-grasses have evolved independent mechanisms for primary iron uptake from the soil. The grasses, which include most of the world’s staple grains, have evolved a distinct ‘chelation’ mechanism to acquire iron from the soil. Strong iron chelators called phytosiderophores (PSs) are synthesized by grasses and secreted into the rhizosphere where they bind and solubilize Fe(III). The Fe(III)-PS complex is then taken up into root cells via transporters specific for the Fe(III)-PS complex. In this study, 31 novel, uncharacterized striped maize mutants available through the Maize Genetics Cooperation Stock Center (MGCSC) were analyzed to determine whether their mutant phenotypes are caused by decreased iron. Many of these proved to be either pale yellow or white striped mutants. Complementation tests were performed by crossing the MGCSC mutants to *ys1* and *ys3* reference mutants. This allowed assignment of 10 *ys1* alleles and 4 *ys3* alleles among the novel mutants. In addition, four *ys^∗^* mutant lines were identified that are not allelic to either *ys1* or *ys3*. Three of these were characterized as being non-allelic to each other and as having low iron in leaves. These represent new genes involved in iron acquisition by maize, and future cloning of these genes may reveal novel aspects of the grass iron acquisition mechanism.

## Introduction

The global demand for crops with high concentrations of nutrients in edible tissues is increasing due to current trends in population growth, global climate change, and decreasing arable land resources (Eckardt et al., 2009). Iron (Fe) deficiency in humans is a global health issue, affecting 1.62 billion people, or about 25% of the world’s population, and it is imperative that we gain a better understanding of the mechanisms that plants use to regulate iron homeostasis, since these will be important targets for future biofortification strategies (McLean et al., 2009; Murgia et al., 2012). Quantitative trait loci (QTL) have been identified for maize grain iron accumulation (Zhang et al., 2017) and identification of additional components of the maize iron homeostatic apparatus may help to elucidate the genes underlying such QTL. Although we have learned a great deal through the study of model organisms such as Arabidopsis, it is important to note that the grasses, which include most of the world’s staple grains, use phytosiderophores (PSs) that are secreted into the rhizosphere where they bind and solubilize Fe(III) (Tagaki, 1976; Tagaki et al., 1984, 1988). PSs are not made or used by non-grass species.

Biofortification of crops has been restricted by our limited knowledge of the molecular mechanisms controlling iron uptake, translocation, accumulation, and deposition in the grain. Attempts to increase iron content have been promising, but these efforts have been focused on the relatively small set of known genes that are involved in iron homeostasis. The iron-storage protein, ferritin (Briat and Lobreaux, 1998; Briat et al., 1999), has been expressed in rice endosperm, to increase iron and zinc content (Goto et al., 1999; Drakakaki et al., 2005). The iron uptake machinery has been a target for biofortification by engineering key enzymes involved in PS synthesis (Higuchi et al., 1999; Takahashi et al., 1999, 2001; Suzuki et al., 2008). These efforts have been only partially successful, suggesting that identifying additional genes involved in mobilization and translocation within the plant could be helpful to develop additional strategies for the production of biofortified crops.

In plants, iron is essential for photosynthesis, respiration, and many other processes, but is only sparingly soluble in aqueous solution, making adequate acquisition by plants a serious challenge (Marschner, 1995). Furthermore, iron is highly reactive and if over-accumulated can cause cellular damage. As a response to these key properties of iron, plants have evolved highly regulated iron mechanisms to ensure efficient and tightly controlled acquisition from the soil. Most plants use a combination of rhizosphere acidification, iron reduction, and uptake via the ZIP (ZRT, IRT-like protein) family transporter, IRT1 (iron-regulated transporter). In this strategy, iron is first solubilized and then taken up from the soil, as reviewed in Walker and Connolly (2008), Jeong and Guerinot (2009), and Morrissey and Guerinot (2009). In contrast, the grasses, which include most of the world’s staple grains, have evolved a distinct ‘chelation’ mechanism to acquire iron from the soil. PSs are synthesized by grasses and secreted into the rhizosphere where they bind and solubilize Fe(III) (Tagaki, 1976; Tagaki et al., 1984, 1988). The Fe(III)-PS complex is then taken up into root cells via transporters specific for the Fe(III)-PS complex (Romheld and Marschner, 1986; von Wiren et al., 1994). This mechanism is also known as ‘Strategy II.’ The Fe(III)-PS uptake transporter Yellow Stripe1 (YS1) has been studied extensively (Curie et al., 2001; Yen et al., 2001; Roberts et al., 2004; Schaaf et al., 2004; Murata et al., 2006; Harada et al., 2007; Inoue et al., 2009; Lee et al., 2009), and is a proton-coupled symporter of Fe(III)-PS complexes (Schaaf et al., 2004).

Phytosiderophores are chemically quite distinct from bacterial and fungal siderophores (Miethke and Marahiel, 2007) and belong to a class of compounds called mugineic acids (Ma and Nomoto, 1996), with a well-worked out biosynthesis (Mori and Nishizawa, 1987; Kawai et al., 1988; Shojima et al., 1990; Ma et al., 1995; Takahashi et al., 1999; Kobayashi et al., 2001). In contrast to the details established for PS biosynthesis and Fe-PS uptake, the molecular details of PS secretion have not been as well-characterized. In several grass species, PSs are secreted according to a diurnal cycle, with release occurring several hours after sunrise (Zhang et al., 1991; Walter et al., 1995; Ma et al., 2003; Reichman and Parker, 2007; Ueno et al., 2007; Nagasaka et al., 2009; Bernards et al., 2014). Large numbers of vesicles have been observed in barley roots just prior to the daily release of PS, suggesting that PSs are secreted by exocytosis (Nishizawa and Mori, 1987; Sakaguchi et al., 1999; Negishi et al., 2002). Furthermore, microarray analysis of barley roots indicated that expression of genes associated with polar vesicle transport increases in the early morning (Negishi et al., 2002). The anion channel blockers anthracene-9-carboxylic acid and phenylglyoxal were shown to inhibit PS secretion by barley roots (Sakaguchi et al., 1999), potentially indicating that anion channels are involved in loading PS to secretory vesicles. Alternatively, anion channels in the plasma membrane (PM) could be responsible for PS transport across the PM. Major facilitator superfamily transporters with PS efflux activity were recently identified in rice and barley, and have been called transporter of mugineic acid (TOM1) (Nozoye et al., 2011).

A classically known mutation in maize called *yellow stripe3* (*ys3*; Wright, 1961) renders plants unable to secrete PSs, even though PSs are synthesized in normal amounts (Basso et al., 1994; Lanfranchi et al., 2002). The *Ys3* gene in maize is located between 85,618,053 and 114,789,459 on chromosome 3 based on two genetic markers (IDP3861 and IDP4688) on the IBM2 2008 Neighbors map. A partial gene with similarity to TOM1 (GRMZM2G063306 also called ZEAMMB73_058478) is located within this interval in the maize reference sequence version 3 but the sequence contained two sequence gaps in the region occupied by GRMZM2G063306. Based on sequence similarity and strong expression during iron deficiency, this gene was suggested as a candidate for the locus affected in *ys3* mutants (Nozoye et al., 2013; Li et al., 2014), but genetic evidence for this assignment has not been presented.

In spite of this progress in understanding the process of PS synthesis, release, and uptake of Fe-PS complexes, there are many gaps in our understanding of what makes a particular grass species or cultivar ‘iron efficient.’ In Kentucky bluegrass, for example, the amount of PS release does not correlate well with resistance to iron deficiency (Buxton et al., 2012). Because of this complexity, we sought to understand the genes in *Zea mays* (maize) that contribute to iron efficiency, by examining the set of maize mutants available through the Maize Genetics Cooperation Stock Center (MGCSC) that have been described as ‘yellow striped’ or ‘green striped.’ Both these descriptions may refer to iron deficiency chlorosis that is typical in both *ys1* and *ys3* maize mutants, and is characterized by yellow interveinal regions and green veins. By performing allelism tests with *ys1* and *ys3* reference mutant plants, we have identified novel yellow striped mutants (that we designate as ys^∗^, pending gene identification and assignment of new nomenclature) that may shed light on additional components contributing to iron efficiency in maize. We further characterized the sequence of GRMZM2G063306 in the WT B73 genome and the *ys3* reference mutants. We have identified four new alleles of *ys3*. Based on the evidence from our sequencing of GRMZM2G063306 in multiple independent *ys3* mutants, we present strong genetic evidence that GRMZM2G063306 (ZmTOM1) corresponds to the *Ys3* gene of maize.

## Materials and Methods

### Plant Material and Growth Conditions

Maize (*Zea mays*) plants of B73 and W22 inbred lines were used as WT reference in our experiments, as indicated in the text. Uncharacterized yellow striped mutants were obtained from the MGCSC^1^.

For all experiments involving genetic crosses, stocks were grown at the University of Massachusetts Crop and Animal Research and Education Center, South Deerfield, MA, United States, during the summer season between May and September. Mutant plants were supplemented with foliar iron (Fe-EDDHA) through growing season to alleviate chlorosis. For the purposes of initial phenotyping, plants were grown in the greenhouse in a 4:1 v/v mix of potting soil and Turface. All phenotyping was also repeated under field conditions. Supplemental light was supplied with high-pressure sodium lamps to give a 20 h light period each day. For quantitative polymerase-chain-reaction (PCR) analysis, plants were grown in a sand:Turface mix (9:1 v/v) irrigated with water until germination and then irrigated with modified Hoagland’s nutrient solutions, with 1 mM KH_2_PO_4_, 3.75 mM KOAc, 5 mM Ca(NO_3_)_2_, 1.25 mM KNO_3_, 2 mM MgSO_4_, 3.75 mM NH_4_OAc, 46 uM H_3_BO_3_, 9.1 uM MnCl_2_, 0.77 uM ZnSO_4_, 0.32 uM CuSO_4_, and 0.83 uM H_2_MoO_4_ (Yordem et al., 2011) containing 100 μM FeSO_4_-EDTA every 48 h. Plants were grown for 10 days after germination before the root tissue was collected.

### PCR and Sequencing of *ZmTOM1* in *ys3* Mutant Lines

Genomic DNA was extracted from leaves of *ys3* mutant plants (*ys3*:*04HI-A632GN-144, ys3*:*67-2403, ys3*:*04HI-Oh43xA632GN-187*, and *ys3*:*07IL-B73GN-279*) and the exons of *ZmTOM1* (GRMZM2G063306/Zm00001d041111) were amplified using primers listed in Supplementary Table 1. Amplifications were performed using ExTaq polymerase (Takara, Madison, WI, United States), with cycling conditions of 95°C, 60 s followed by 35 cycles of 95°C, 15 s, 55°C, 30 s, and 72°C, 60 s, with a final extension step at 72°C for 5 min. PCR products were gel purified before sequencing.

### Real-Time PCR Analysis (qRT-PCR)

The root tissue was flash frozen in liquid nitrogen after harvesting. The frozen root tissue was ground using a Tissuelyser (QIAGEN, Valencia, CA, United States) in 2 ml tubes containing 3.2 mm chrome steel beads (BioSpect Products, Bartlesville, OK, United States). Total RNA was extracted using QIAGENR^®^Neasy Plant Mini Kit (QIAGEN, Valencia, CA, United States), and on-column DNAse treatment step was included for all samples. cDNA was synthesized from 750 ng of total RNA using SuperScript IV VILO (Life Technologies, Carlsbad, CA, United States). For real-time PCR (RT-PCR) analysis, Quantprime primer design webtool (Arvidsson et al., 2008) was used to design *ZmTOM1* primers. The primer efficiency of each set of primers (Supplementary Table 2) was evaluated empirically by serial dilution curve of cDNA. PowerUP^TM^ SYBR^TM^Green Master Mix (Life Technologies, Carlsbad, CA, United States) was used in quantitative RT-PCR experiments. A two-step PCR protocol was used with the following conditions: initial cycle of 50°C, 120 s, and 95°C, 120 s, and 40 cycles of 95°C, 15 s, and 60°C, 60 s. After two-step cycling was completed, melting curve was performed to ensure that single amplicon was obtained from each reaction. To determine transcript levels, the threshold cycle (*C*_t_) values from target gene was normalized to *ZmGAPDH* reference gene for each sample and by the ΔΔ*C*_t_ method, we calculated fold change compared to B73 WT. Data represent three biological replicates.

### Inverse PCR

Genomic DNA (∼1 μg) was digested with *Aci*I and *Nla*III (New England Biolabs) for 2.5 h at 37°C and reaction was stopped by incubating for 20 min at 65°C. The DNA was then diluted 25-fold, and ligation was performed using 20 units of Epicenter^®^T4 DNA ligase (Illumina, Inc., Madison, WI, United States) overnight at either 20°C for blunt ends or 4°C for sticky ends. The resulting ligation was purified using phenol/chloroform (1:1, v/v) and ethanol precipitation in the presence of 40 μg of glycogen. Then, 1/6 of the purified ligation was used as template for the 1^st^ round of PCR, with primers oZmTOM1_4456 and oZmTOM1_4504 for *Aci*I restriction digest, or primers oZmTOM1_3641 and oZmTOM1_5012 for *Nla*III restriction digestions. The 2^nd^ round of PCR was performed using 1 ul of a 1:100 dilution of the PCR product from the 1^st^ round as template using nested primers oZmTOM1_4338 and oZmTOM1_4573for *Aci*I digested DNA or oZmTOM1 4774 and oZmTom1_5154, for *Nla*III digested DNA. Amplifications were performed using ExTaq polymerase (Takara, Madison, WI, United States), with cycling conditions of 95°C, 2 min, and 25 cycles of 95°C, 15 s, 57°C, 30 s, 72°C for 2 min, and a final elongation step for 10 min. Primer sequences for inverse PCR (iPCR) are listed in Supplementary Table 3.

### Metal Measurement

Leaves of at least 10 individual plants were collected from 19-day-old plants grown in the greenhouse and samples were dried at 65°C for 72 h. In every experiment, all controls and mutants were grown simultaneously and using the same soil batch. Metal concentrations were determined by inductively couple plasma mass spectrometry (ICP-MS) at the Donald Danforth Plant Research Institute.

## Results

### Complementation Testing of Yellow Striped Mutants from MGCSC

We obtained 31 mutants classified as having a yellow striped phenotype from the MGCSC (**Table 1**). These were planted in the field and phenotypic analysis indicated that 21 of the lines showed the phenotype typical of iron deficiency chlorosis. In the other 10 lines, we either did not observed stripes at all or else observed a solid yellow or white striped phenotype (**Table 1**). To identify new genes involved in iron uptake in maize, and to identify new alleles for *ys3*, we performed complementation tests between the uncharacterized yellow striped mutants and the reference maize mutants *ys1:ref* and *ys3:ref*. Due to stunting or sterility of some mutant stocks, not all crosses were obtained. From these crosses, we identified 10 new alleles for *ys1* and 4 new alleles for *ys3*. Moreover, we found four novel yellow stripe mutants, *ys^∗^-PI262172, ys^∗^*:*N2398, ys^∗^*:*PI228180*, and *ys^∗^*:*04HI-A632XOh43GN-18*, that are not allelic to *ys1* or *ys3*, and thus may represent new maize genes involved in iron uptake or homeostasis.

**Table 1:** Yellow or green striped mutants from the Maize Genetics Cooperation Stock Center (MGCSC) and results of complementation tests with *ys3:ref* and *ys1:ref*.

MGCSC	Mutation name	Phenotype	Allelic to	Allelic to
number			*ys3:ref*	*ys1:ref*
503A	*ys1:ref*	Chlorotic, often sterile	–	–
503B				
3812I	*ys1-N2261*	Small, chlorotic	No	Yes
6003B	*ys1-8912*	Small, chlorotic	No	Yes
6003G	*ys1-PI267219*	Small plant, chlorotic	No	Yes
6003I	*ys1-5-8(5575)*	Small plant, chlorotic	No	Yes
3812C	*ys1-N139B*	Small, severely chlorotic	No	Yes
6003L	*ys1-03HI-B73GN-182*	Chlorotic	No	Yes
6505A	*ys1-03HI-B73xMo17GN-210*	Small plant, chlorotic	NT	Yes
6505C	*ys1-04HI-A632xOh43GN-137*	Yellow striped plants also had crinkled leaves	No	Yes
3812B	*ys1-N71B*	Chlorotic, asynchronous flowering	No	Yes
304A	*ys3:ref*	Chlorotic, sometimes sterile	–	–
311F				
311G				
6505E	*ys3-07IL-B73GN-279*	Chlorotic	Yes	No
6003C	*ys3-67-2403*	Small, chlorotic	Yes	No
6412B	*ys3-04HI-A632GN-144*	Small plant, chlorotic	Yes	No
6505D	*ys3-04HI-Oh43xA632GN-187*	Chlorotic	Yes	No
3812O	*ys^∗^-N2398*	Chlorotic, chlorosis easily reversed by spraying leaves with Fe	No	No
6003D	*ys^∗^-PI228180*	Chlorotic, chlorosis easily reversed by spraying leaves with Fe. Yellow stripes appear again at time of flowering	No	No
6505B	*ys^∗^-04HI-A632xOh43GN-18*	Small plant, chlorotic	No	No
3812D	*ys-N326A*	Pale yellow	NT	NT
3812G	*ys-N634B*	Pale yellow	NT	NT
3812H	*ys-N2000*	Pale yellow	NT	NT
3812J	*ys-N2268*	Oil yellow – not striped	No	No
3812K	*ys-N2300*	White striped	NT	NT
3812M	*ys-N2361*	No yellow or striped phenotype observed	NT	NT
3812N	*ys-N2379*	No yellow or striped phenotype observed	NT	NT
6003K	*ys-03HI-B73xMo17GN-1123*	Poor germination; stripes not observed	NT	NT
6005L	*yel-str-W23*	Pale yellow	NT	NT
6003F	*ys-PI262475*	Small plant, yellow, not striped	No	No
6003A	*ys1-1479*	Chlorotic	No	Yes
6003E	*ys^∗^-PI262172*	Stunted, chlorotic	No	No
6003J	*ys?-68-1354*	Small plant, chlorotic	No	NT
6412C	*ys?-07IL-B73GN-171*	Yellow striped plants died	NT	No
3812L	*ys?-N2303*	Small, chlorotic, poor fertility	No	NT

*NT indicates that allelism was not tested.*

### Analysis of ZmTOM1 Coding Sequence in *ys3* Alleles

**FIGURE 1:**
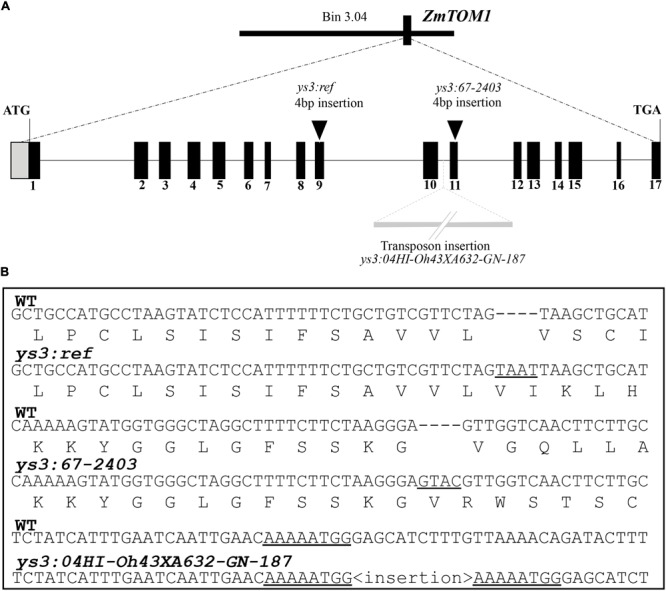
*ZmTOM1* sequence analysis. **(A)** Overview of the *ys3* locus and gene model of *ZmTOM1* showing *ys3* mutant alleles. Exons are indicated by black boxes and the positions of mutations are indicated by black triangles. **(B)** Genomic sequences and predicted translation of *ys3:ref* and *ys3:67-2403* alleles. WT sequence is shown for comparison. For *ys3:04HI-Oh43XA632-GN-187*, genomic sequence of the site of insertion is shown, with the 8 bp direct repeat flanking the insertion underlined.

**FIGURE 2:**
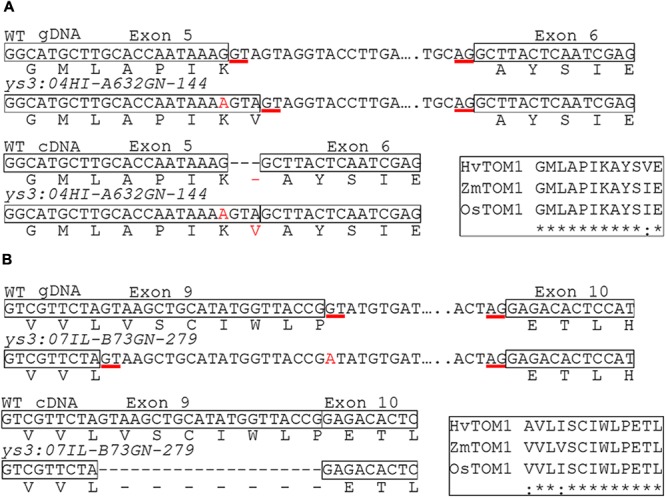
*ys3* mutations causing altered splicing. Boxes indicate exon sequences. Nucleotide changes relative to B73 reference sequence are indicated in red. Red underlined sequences are the splice donor and acceptor sites used in WT and the two mutant *ys3* alleles. Genomic DNA and cDNA sequences with their predicted translation are shown. **(A)** *ys3*:*04HI-A632GN-144* and **(B)** *ys3*:*07IL-B73GN-279* alleles. The inset rectangles show alignments of the corresponding amino acid sequences of transporter of mugineic acid (TOM1) from barley, rice, and maize showing conserved regions. ^∗^ indicates an amino acid identical in all three species and indicates an amino acid that is conserved in all three species.

Because a gene with similarity to TOM1 (GRMZM2G063306 also called ZEAMMB73_058478, here designated *ZmTOM1*) is located within the genomic interval containing *Ys3*, this gene has been suggested as a candidate for the locus affected in *ys3* mutants (Nozoye et al., 2013; Li et al., 2014). To determine whether the suggested candidate gene, ZmTOM1, underlies the long-known *ys3* mutant, we sequenced the exons of *ZmTOM1* in all five alleles of *ys3* (reference allele and the four novel alleles identified through complementation tests; **Figure 1**) to identify causative mutations. The MGCSC holds three stocks designated as *ys3:ref* mutants (304A, 311F, and 311G). In all three lines, *ZmTOM1* sequences were identical, and contained a 4 bp insertion in exon 9. This insertion causes a frame shift followed by 11 novel amino acids before introducing a premature stop codon (**Figure 1**). We found a different 4 bp insertion in exon 11 of *ZmTOM1* in the *ys3*:*67-2403* allele (**Figure 1**). This 4 bp insertion causes a frame shift followed by 107 new amino acids before a stop codon occurs to terminate the protein prematurely. For the *ys3*:*04HI-Oh43XA632-GN-187* allele, we could not amplify fragments containing exon 10 and 11, but partial sequences from both exons could be obtained. We hypothesized that an insertion could be present between these two exons causing failure to amplify that region. Using iPCR, we identified both left and right borders of an insertion containing the characteristic elements of a transposon. The inserted sequences were flanked by 8 bp direct repeats and contained 130 bp terminal inverted repeats (TIRs). We aligned the TIR sequences with the maize reference sequence and identified two regions in chromosome 7 annotated as Far1-related sequence 5, which corresponds to a mutator-like transposable element (MULE). MULE transposons generate 8–10 bp target sequence duplications and have TIRs of >100 bp. Thus, the insertion has all the elements expected for a MULE transposon inserted in the *ys3*:*04HI-Oh43XA632-GN-187* allele (**Figure 1**). A one nucleotide change at the exon–intron border for exon 5 of *ys3*:*04HI-A632GN-144* was observed. Likewise, a one nucleotide change near the 3′ end of exon 9 was observed in *ys3*:*07IL-B73GN-279*. We hypothesized that splicing could be affected these two alleles, and so investigated *ZmTOM1* gene expression in the roots of these plants using Q-RT-PCR. Expression of *ZmTOM1* was observed in both *ys3*:*04HI-A632GN-144* and *ys3*:*07IL-B73GN-279* (data not shown). Since the *ZmTOM1* transcript was observed, we speculated that altered splicing due to the mutations might be leading to aberrant *ZmTOM1* mRNA, so we sequenced the full-length cDNA from each mutant line to test this. We confirmed that the one nucleotide change at the *ys3*:*04HI-A632GN-144* exon–intron junction altered the splice donor site. The mutation causes splicing to occur at a new donor site 3 nucleotides into the adjacent intron (**Figure 2**). As a result, one additional amino acid is inserted without affecting the reading frame. The amino acid is inserted in a strongly conserved region that could lead to a non-functional protein. In the *ys3*:*07IL-B73GN-279* allele, the single nucleotide change occurred at the first nucleotide of intron 9, changing the splice donor site from GT to AT. In the mRNA produced by this allele, a new splice donor site is recognized in exon 9, 21 nucleotides upstream from the original donor site (**Figure 2**). The resulting amino acid sequence is thus missing seven residues in a strongly conserved region. Our results show clear genetic evidence that the *Ys3* gene is *ZmTOM1*.

### Analysis of Novel Yellow Striped Maize Mutants

**FIGURE 3:**
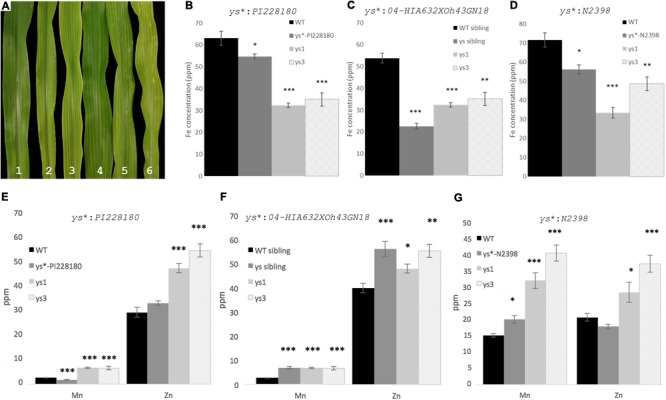
*ys^∗^* mutant phenotypes and iron concentration. **(A)** Leaves of 12-day-old greenhouse grown plants. (1) WT; (2) *ys^∗^:N2398*; (3) *ys^∗^:04HI-A632xOh43GN-18*; (4) *ys^∗^:PI228180*; (5) *ys1:ref*; and (6) *ys3:ref*. Inductively couple plasma mass spectrometry (ICP-MS) measurement of the iron content of **(B)** *ys^∗^:PI228180*, **(C)** *ys^∗^:04HI-A632xOh43GN-18*, and **(D)** *ys^∗^:N2398*. ICP-MS measurements of the Mn and Zn content of **(E)** *ys^∗^:PI228180*, **(F)** *ys^∗^:04HI-A632xOh43GN-18*, and **(G)** *ys^∗^:N2398*. Error bars represent the standard error of the mean (*n* = 10). Significance was addressed using the Mann–Whitney test. Asterisk indicates significant difference from WT (^∗^*p* < 0.05, ^∗∗^*p* < 0.001, ^∗∗∗^*p* < 0.0001).

To evaluate whether the yellow striped phenotype in *ys^∗^* mutants is due to low iron, we analyzed metal levels in leaves of three of the mutants. The *ys^∗^-PI262172* mutant was not included in this analysis, because its stunted growth prevented our obtaining sufficient material for this experiment. Visual inspection of the leaves of 12-day-old WT and mutant plants indicates differences in the severity of the observed chlorosis, with ys^∗^:PI228180 having very mild chlorosis and *ys^∗^:04HI-A632xOh43GN-18, ys1:ref*, and *ys3:ref* having the most marked chlorosis (**Figure 3**). In all three *ys^∗^* mutants tested, the levels of iron were significantly lower than WT (**Figure 3**) indicating that the plants are iron-deficient. Control *ys1* and *ys3* plants are also low in iron, as expected. In a segregating population of *ys^∗^:04-04HI-A632xOh43GN-18* mutants, the iron concentration in yellow striped siblings was less than half (42%) that of WT siblings. The iron concentration in *ys^∗^*:*04HI-A632xOh43GN-18* was significantly lower even than *ys1* and *ys3*, indicating a very substantial alteration in iron homeostasis in these plants. For *ys^∗^*:*PI228180* and *ys^∗^*:*N2398*, iron levels were higher than either *ys1* or *ys3*, but were still significantly lower than the amount in WT control plants.

We also measured the Zn and Mn concentration in the leaves of the mutant plants (**Figure 3**). We note that altered iron homeostasis often causes alterations to multiple metals. For example, *ys1* and *ys3* mutants, which are clearly impaired in iron uptake, have higher Zn and Mn than WT control plants (**Figure 3**). It is possible that this occurs because PS secretion or uptake directly affects Mn and Zn uptake or translocation, but it is also possible that the mechanism is indirect. Like *ys1* and *ys3* mutants, *ys^∗^:04HI-A632xOh43GN-18* and *ys^∗^:N2398* plants have higher Mn and Zn than WT control plants. For *ys^∗^:PI228180*, the Zn concentration in leaves is not significantly different from WT control plants, and the Mn concentration is slightly but significantly lower than that of the WT controls, and much lower that the Mn concentration in the *ys1* and *ys3* mutants. We note that the soil batch used for growth of the *ys^∗^:04HI-A632xOh43GN-18* and *ys^∗^:N2398* plants and their controls was different from the batch used to grow *ys^∗^:PI228180* and its controls.

### Complementation Tests among ys^∗^ Mutants

We performed crosses among three of the four identified *ys^∗^* mutants to determine how many loci are represented by these three mutants. The *ys^∗^-PI262172* mutant was not included in this analysis, because its stunted growth prevented our obtaining the appropriate crosses. F1 seeds were grown in the greenhouse and the phenotypes were recorded. We found complementation among all crosses performed in *ys*^∗^ mutants, indicating that they do not represent alleles. These results show that we have identified three novel genes involved in iron homeostasis (**Table 2**).

**Table 2:** Complementation test results among ys^∗^ mutants.

MGCSC number	Mutation name	Allelic to *ys^∗^*:*N2398*	Allelic to *ys^∗^:04HI-A632xOh43GN-18*	Allelic to *ys^∗^:PI228180*
3812O	*ys^∗^:N2398*	–	No	No
6505B	*ys^∗^:04HI-A632xOh43GN-18*	No	–	No
6003D	*ys^∗^:PI228180*	No	No	–

## Discussion

### The Rationale for Gene Discovery in *Zea mays*

Much of the molecular work on iron uptake and homeostasis in grasses has been performed using rice, both because of its properties as a model organism and also because of the fundamental importance of this species as a crop. Still, the Fe(III)-PS uptake transporter, YS1, was first identified in maize by making use of the excellent genetic resources available in this species (Curie et al., 2001). The YS1 gene has been used directly as a strategy for engineering biofortification with mixed results. In an early study using constitutive expression of barley YS1 in rice, plants showed superior growth in alkaline soil conditions but did not contain significantly more iron in grains (Gomez-Galera et al., 2012). Later, barley YS1 expressed in rice was shown to promote the preferential mobilization and loading of Fe in seeds while displacing Cd and Cu (Banakar et al., 2017). At present, two key uptake genes, *Ys1 and Ys3* (*TOM1*), for the grass specific mechanism are understood, as are the genes involved in PS synthesis, but it is unclear whether additional grass specific components exist. If they do, they will need to be discovered directly in grass species such as maize.

### Identification of the *Ys3* Gene

Maize *ys3* mutants lack the ability to secrete PS (Lanfranchi et al., 2002). *ZmTOM1* has been proposed as candidate gene for *Ys3* because of its function as PS effluxer (Nozoye et al., 2011) and its location within the same map interval as the genetically identified *ys3* mutant allele. Previous reports analyzing the *ys3* transcriptome during iron deficiency suggested reduced expression and alternative splicing of *ZmTOM1* (Nozoye et al., 2013). However, this approach could not definitively assign *ZmTOM1* as the *Ys3* gene, since other genes (*ZmMATE3/ZmPEZ1*) had reduced expression in *ys3* mutants, and mutations in *ZmTOM1* were not identified. Here, we were able to show that multiple alleles of *ys3* could be found among the yellow striped mutants held at the MGCSC, and that each of these carries a unique mutation that is expected to abolish the function of the ZmTOM1 protein.

### Three Novel Yellow Striped Maize Mutants

In this study, we identified three novel yellow striped mutants whose phenotype is apparently caused by low iron content. These mutants represent three different loci involved in iron homeostasis. Genetic mapping to identify the underlying genes responsible for the yellow striped phenotype in these mutants will reveal unknown elements of the iron homeostasis machinery and may provide new options for biofortification. Initially, it appeared as though our screening of the MGCSC mutant collection had reached saturation since multiple alleles for both *ys1* and *ys3* were obtained. However, three novel loci contributing to iron content in leaves were identified, indicating that saturation mutagenesis has likely not been reached and additional genes causing an iron deficiency induced yellow striped phenotypes in maize could be uncovered. Genetic mapping of the three ys^∗^ mutants is underway to discover the genes responsible for these interesting metal homeostasis phenotypes. Future work will also include tests to indicate whether additional iron supply or direct iron supply to the leaves can alleviate the ys^∗^ phenotypes, and tests of the iron concentration in grains of the mutant plants to see whether the grain concentration of iron is altered in the mutants.

## Author Contributions

EW conceived the project, was responsible for the experimental design, and also performed some of the genetics crosses and phenotyping in the field. DC-R conducted screening and genetics crosses and performed all of the molecular work on the project.

## Conflict of Interest Statement

The authors declare that the research was conducted in the absence of any commercial or financial relationships that could be construed as a potential conflict of interest.

## Footnotes

1 http://maizecoop.cropsci.uiuc.edu
