# Supplementary material for: Analysis of Yellow Striped Mutants of Zea mays Reveals Novel Loci Contributing to Iron Deficiency Chlorosis
Source: Front Plant Sci. 2018 Feb 20;9:157. doi: 10.3389/fpls.2018.00157 (PMC5826256; doi:10.3389/fpls.2018.00157)
Supplement: Supplementary file 1 [file Table_1.DOCX]

Supplementary Table 1. Primer sequences used to analyze *ZmTOM1*

| Exon Coverage | Primer ID | Primer sequence 5’-3’ |
| --- | --- | --- |
| Exon 1 | oZmTom1_17  oZmTom1_437 | TGCTAAACCACCAAGGTCGAA  TATTTTGTGCGAGGGGAGATG |
| Exon 2-4 | oZmTom1_1871  oZmTom1_2577 | GCTGCTTACATGCACCATGC  GTGCAAGCATGCCATTGAGAG |
| Exon 2-6 | oZmTom1_1871  oZmTom1_2842 | GCTGCTTACATGCACCATGC  ACGTTATTAATCTCAACTCGAGCAA |
| Exon 5-6 | ZmTom1_2310  oZmTom1_2842 | GGTCTCGTTCTTCTGGGGTGT  ACGTTATTAATCTCAACTCGAGCAA |
| Exon 6-7 | oZmTom1_2626  Reverse to Genomic2 | GCCATGGCAGTTAGTTTCAAAT  TTGGATATTGCCGTGCAG |
| Exon 8-9 | oZmTom1_2878  oZmTOM1_3686 | CTTATTGTTGGCCCGTCCATT  AAGGATCAGCGCATCCTAACTTC |
| Exon 6-9 | oZmTom1_2626  oZmTom1_3485 | GCCATGGCAGTTAGTTTCAAAT  CCCAAAGGGACACAATCTTCTTC |
| Exon 10 | oZmTom1_4146  oZmTom1-3194 | GGGAGCATGTTTACCGATTGA  AGCGTGAAAACACAGTACGC |
| Exon 11 | oZmTOM1_4697  cDNA 690-670 | TTGAACAAAAATGGGAGCATCTTTGT  TGTGGACTGGCCTGTAGATG |
| Exon 10-11 | oZmTom1_4146  oZmTom1_4882 | GGGAGCATGTTTACCGATTGA  CAACGTCTGGTAGCAGCTATTGT |
| Exon 12-14 | oZmTom1_5154  oZmTom1_5962 | ACACGGTCTCAAGAGCCACAA  TGCTGTGGCTGTTTTCAAAGG |
| Exon 12-15 | oZmTom1_5154  oZmTom1_6071 | ACACGGTCTCAAGAGCCACAA  TATATGATGCGTGGGGGACAATA |
| Exon 15-17 | oZmTom1_5833  oZmTom1_6822 | TGCAGAACTATGCTGTGGCAAG  ATTCCATTGCTTGGGTTCGAG |
| Exon 16-17 | oZmTOM1_6237  oZmTOM1_6882 | CCAGATTCGTGTGCACCATTATT  TCCGTTGTATGCCACTCAAAAAC |
|  | oZmTOM1_67 | GTAGCGTGTCCCTGTCCATTGTA |
|  | oZmTOM1_4515 | GTTTGTGGAGTCCTTGAGTGTCC |
|  | oZmTOM1_3161 | CACGGCAATATCCAAACGTATTC |
