# Supplementary material for: Analysis of Yellow Striped Mutants of Zea mays Reveals Novel Loci Contributing to Iron Deficiency Chlorosis
Source: Front Plant Sci. 2018 Feb 20;9:157. doi: 10.3389/fpls.2018.00157 (PMC5826256; doi:10.3389/fpls.2018.00157)
Supplement: Supplementary file 2 [file Table_2.DOCX]

Supplementary Table 2. Primers sequence used in Q-RT-PCR.

| Primer ID | Primer sequence 5’-3’ |
| --- | --- |
| oZmTOM1_qPCR_Fw | GCTTGCACCAATAAAGGCTTACTC |
| oZmTOM1_qPCR_Rv | CCAATGGACGGGCCAACAATAAG |
| ZmGADPH_Fw | CCTGCTTCTCATGGATGGTT |
| ZmGADPH_Rv | TGGTAGCAGGAAGGGAAACA |
