# Supplementary material for: Analysis of Yellow Striped Mutants of Zea mays Reveals Novel Loci Contributing to Iron Deficiency Chlorosis
Source: Front Plant Sci. 2018 Feb 20;9:157. doi: 10.3389/fpls.2018.00157 (PMC5826256; doi:10.3389/fpls.2018.00157)
Supplement: Supplementary file 3 [file Table_3.DOCX]

Supplementary Table 3. Primers used for inverse PCR

| Primer ID | Primer sequence 5’-3’ | PCR round | Restriction enzyme |
| --- | --- | --- | --- |
| oZmTOM1_4456 | TGGAGTGTCTCCTAGTAACAAAATGG | 1^st^ | *Aci*I |
| oZmTOM1_4504 | GGACTCCACAAACAGAAGACGTA | 1^st^ | *Aci*I |
| oZmTOM1_4338 | GCCAAGAATCGTTTGTAGAAATTCA | 2^nd^ | *Aci*I |
| oZmTOM1_4573 | TCATTGCGTACTGTGTTTTCACG | 2^nd^ | *Aci*I |
| 3641..3620 | CAAATGAACATACCTGCAATC | 1^st^ | *Nla*III |
| oZmTOM1_5012 | TGTTATTTTTCCTCAACCCAGGA | 1^st^ | *Nla*III |
| oZmTOM1 4774 | GCCCACCATACTTTTTGTCACTT | 2^nd^ | *Nla*III |
| oZmTom1_5154 | ACACGGTCTCAAGAGCCACAA | 2^nd^ | *Nla*III |
